# Laser-activated autologous adipose tissue-derived stromal vascular fraction restores spinal cord architecture and function in multiple sclerosis cat model

**DOI:** 10.1186/s13287-022-03222-2

**Published:** 2023-01-11

**Authors:** Mariam F. Farid, Yara S. Abouelela, Noha A. E. Yasin, Asmaa K. Al-Mokaddem, Abdelbary Prince, Marwa A. Ibrahim, Hamdy Rizk

**Affiliations:** 1 Department of Anatomy and Embryology, Faculty of Veterinary Medicine, Cairo University, Giza, 12211 Egypt; 2 Department of Cytology and Histology, Faculty of Veterinary Medicine, Cairo University, Giza, Egypt; 3 Department of Pathology, Faculty of Veterinary Medicine, Cairo University, Giza, Egypt; 4 Department of Biochemistry and Molecular Biology, Faculty of Veterinary Medicine, Cairo University, Giza, Egypt; 5 Department of Biomedical Research, Armed Forces College of Medicine, Cairo, 12211 Egypt

**Keywords:** Multiple sclerosis, Stromal vascular fraction, Mesenchymal stem cells, Flow cytometry, MRI, Immunohistochemistry, TEM

## Abstract

**Background:**

Multiple sclerosis (MS) is the most frequent non-traumatic neurological debilitating disease among young adults with no cure. Over recent decades, efforts to treat neurodegenerative diseases have shifted to regenerative cell therapy. Adipose tissue-derived stromal vascular fraction (SVF) comprises a heterogeneous cell population, considered an easily accessible source of MSCs with therapeutic potential in autoimmune diseases. This study aimed to assess the regenerative capacity of low-level laser-activated SVF in an MS cat model.

**Methods:**

Fifteen adult Persian cats were used in this study: Group I (control negative group, normal cats), Group II (EB-treated group, induced for MS by ethidium bromide (EB) intrathecal injection), and Group III (SVF co-treated group, induced for MS then treated with SVF on day 14 post-induction). The SVF was obtained after digesting the adipose tissue with collagenase type I and injecting it intrathecal through the foramen magnum.

**Results:**

The results showed that the pelvic limb’s weight-bearing locomotion activity was significantly (*P* ≤ 0.05) recovered in Group III, and the Basso, Beattie, and Bresnahan (BBB) scores of hindlimb locomotion were significantly higher in Group III (14 ± 0.44) than Group II (4 ± 0.31). The lesion’s extent and intensity were reduced in the magnetic resonance imaging (MRI) of Group III. Besides, the same group showed a significant increase in the expression of neurotrophic factors: BDNF, SDF and NGF (0.61 ± 0.01, 0.51 ± 0.01 and 0.67 ± 0.01, respectively) compared with Group II (0.33 ± 0.01, 0.36 ± 0.006 and 0.2 ± 0.01, respectively). Furthermore, SVF co-treated group revealed a significant (*P* ≤ 0.05) increase in oligodendrocyte transcription factor (Olig2) and myelin basic protein (4 ± 0.35 and 6 ± 0.45, respectively) that was decreased in group II (1.8 ± 0.22 and 2.9 ± 0.20, respectively). Moreover, group III showed a significant (*P* ≤ 0.05) reduction in Bax and glial fibrillary acidic protein (4 ± 0.53 and 3.8 ± 0.52, respectively) as compared with group II (10.7 ± 0.49 and 8.7 ± 0.78, respectively). The transmission electron microscopy demonstrated regular more compact, and markedly (*P* ≤ 0.05) thicker myelin sheaths (mm) in Group III (0.3 ± 0.006) as compared with group II (0.1 ± 0.004). Based on our results, the SVF co-treated group revealed remyelination and regeneration capacity with a reduction in apoptosis and axonal degeneration.

**Conclusion:**

SVF is considered an easy, valuable, and promising therapeutic approach for treating spinal cord injuries, particularly MS.

**Supplementary Information:**

The online version contains supplementary material available at 10.1186/s13287-022-03222-2.

## Background

Spinal cord injuries in mammals cause degenerative neurons and axons, resulting in significant sensorimotor dysfunction, paraplegia, or tetraplegia [1, 2]. Multiple sclerosis (MS) remains an idiopathic, autoimmune, chronic inflammatory demyelinating disorder that destroys the oligodendrocytes accountable for the myelin sheath formation in the CNS [3–5]. Pathologically, MS is characterized by perivascular inflammatory infiltrates containing T and B lymphocytes, besides antibodies against myelin [6]. Otherwise, recent medications are limited to controlling immune responses that may only be useful in the early stages of the disease [5, 7]. Rehabilitation that depends on the use of regenerative stem cell medicines has generated a new promise in supporting spinal cord function–structural recovery [1, 8] in dogs [9, 10], monkeys [11, 12], rats [13, 14], and rabbits [15, 16]. Mesenchymal stem cells (MSCs) can be harvested and processed easily from adipose tissues with minimal invasion and infection, giving rise to oligodendrocytes and neural progenitor cells [2, 17]. Additionally, MSCs have an anti-inflammatory activity that decreases cytokine and neuronal cell death and secretes neurotrophic factors that promote regeneration [18, 19].

The stromal vascular fraction (SVF) is a by-product of adipose tissue harvesting and digestion. It consists of various non-expanded cells, including adipose mesenchymal stem cells (ASCs), adipocytes, T regulatory cells, macrophages, endothelial precursor cells, and numerous leukocytes [20, 21]. The proportion of ADSCs in SVF varies between 40 and 50%, depending on the harvesting and digestion method.

While considerable effort in treating spinal cord injury focuses on small laboratory animals, large animal models are needed to properly evaluate stem cell therapy’s safety and clinical efficacy after spinal cord injury [22].

Therefore, our study is designed to evaluate the therapeutic potential of autogenic Low laser-activated SVF transplantation in the functional recovery and structural remodeling in the ethidium bromide-induced MS in cats by magnetic resonance imaging (MRI), histopathological and immunohistochemical examination, transmission electron microscopy, and gene expression analysis by RT-PCR.

## Methods

### Study design

The Veterinary Medicine Cairo University Institutional Animal Care and Use Committee approved all experimental animal protocols (Vet-CU-IACUC) with approval number Vet Cu12/10/2021/392, as well as shelters approval sheets were applicable for this study.

### Experimental animals and groups

Fifteen male adult Persian cats (2–3 years) were collected from different shelters around Giza and housed in the Faculty of Veterinary Medicine, Anatomy Department with a minimum acclimation period of one week before major surgery. Food and water were available *ad libitum* at ideal room temperature (20–23 °C). All cats were evaluated before the study to exclude any animals suffering from nervous manifestations. The animals were grouped into three (n = 5). Group I (control negative group, normal cats), Group II (EB-treated group, induced for MS by Ethidium bromide intrathecal injection in the thoracolumbar region), and Group III (SVF co-treated group, induced for MS then treated with SVF on day 14 post-induction). Sample collection was applied 28 days post-SVF injection in all groups.

### Induction of demyelination

The spinal cord demyelination was performed in groups (Gp II and Gp III) using ethidium bromide (Suvchem Laboratory Chemicals) [23, 24]. The cats were anaesthetized using xylazine 1 mg/kg (Xyla-Ject® 2% ADWIA Co., A.R.E.) and Ketamine 5% I/M 10 mg/kg (Ketamar® 5% Sol. Amoun Co. A.R.E) [23]. They were positioned on the sternal recumbency, and a dorsal midline incision was applied along the T12 to L2. The subcutaneous fascia was dissected until the lumbodorsal fascia was reached, and the supraspinous ligament was incised around the spinous process and on the midline. The multifidus lumborum muscle was bluntly removed to expose the dorsal laminae of L1. Two bilateral holes were drilled using a dental drill with a rounded bur diameter of 1.2 mm through the dorsal lamina of L1. A single injection of 6 μl 0.1% EB was injected into each hole using a 10-μl microsyringe [25].

The wound was sutured with a simple continuous pattern for muscles and subcutaneous tissues using Vicryl size 3–0 and silk size 2–0 for the skin in a single interrupted pattern. Animals were injected with a systemic course of antibiotics for 5 days: analgesics (Meloxicam® EL Nasr Co, A.R.E, 0.2 mg/Kg SC for 3–4 days) and daily dressing to the wound.

Fourteen days after induction, animals were administered either a single dose of normal saline (Group II) or laser-activated SVF intrathecally through the foramen magnum (Gp III).

### Adipose tissue collection

On day 14 post-induction, under general anesthesia, a small skin incision in the inguinal region was aseptically operated, and adipose tissue (~ 20 g) was collected from S/C tissue in a 50-ml sterile falcon tube.

### Tissue processing and isolation of SVF

The tissue was processed following a sterile protocol in the laminar airflow. The collected fat was chopped into small pieces, washed thrice with phosphate-buffered saline, and then added an equal volume of 0.1% collagenase type I into it. The tissue was incubated in a rotary incubator at 37 °C, with constant agitation for one hour. After digestion, an equal volume of DMEM containing 10% fetal bovine serum was added to neutralize the collagenase. The SVF was centrifuged at 800 g for 10 minutes to separate the collagenase [26]. Then, the SVF was washed thrice with phosphate buffer saline to eliminate residual collagenase. After the last round of centrifugation, cells were stained with trypan blue and counted using a hemocytometer.

### In vitro SVF expansion

DMEM was placed ten times the cell sedimentation volume in a centrifuge tube. The cells were then inoculated at a density of 30%–50% into a culture flask, followed by adding a complete medium to a final volume of 10 ml; the flasks were placed in an incubator at 37 °C with 5% CO_2_. After 24 h, the medium was replaced to remove non-adherent cells; half of the medium was replaced every 2 days until the cells reached a confluency of 80%–90%. The offspring produced in this step was referred to as the first passage (P1) cells after 7–12 days. Then, 1 × 10^6^ cells were inoculated for 4 days in the culture medium (second passage) [27].

### Flow cytometry

Following the second passage, stem cells were harvested. Cells were treated with a 10% trypsin EDTA solution for 5–10 min in the incubator, followed by a wash. The cell pellet was then incubated for one hour with 1% bovine serum albumin containing primary antibodies against the following cell surface markers: CD 34, CD73, CD45, CD44, and CD105. The cells were then incubated for 30 min with the secondary antibody before immunophenotyping using a fluorescence-activated cell sorting cell analyzer [28].

### Adipogenic differentiation

After the third passage, the cells were trypsinized and placed at 10^7^ cells per plate. Then, they were differentiated into chondrogenic and adipogenic lineages to demonstrate the isolated cell’s mesenchymal phenotypes.

The adipogenic medium containing 100 nM dexamethasone, 50 mg/ml indomethacin, and 100 ML ascorbic acid, was added to each well and changed every 3 days to induce adipogenesis. After 21 d, the culture medium was removed, and the cells were fixed with 4% formalin at room temperature for one hour before being stained with Oil Red O solution in 10% isopropanol for 15 min. The adipose droplets were visualized using a light microscope [29].

### Chondrogenic differentiation

To induce chondrogenesis, the media that contained Dulbecco’s modified Eagle’s medium with 1% fetal bovine serum, 6.25 lg/ml insulin (Sigma), 10 ng/ml transforming growth factor-b1 (Sigma), and 6.25 lg/ml transferrin (Sigma) was added to each flask. The media was changed every 3 days. Chondrogenic differentiation was assessed via Safranin-O staining. [29].

### Injection of SVF

The stem cell preparation (10 × 10^6^ nucleated cells) was activated using a red laser diode (635 nm) for 10 minutes at 7 cm, then injected directly into the foramen magnum on day 14 post-induction of the MS as declared by [9].

Groups I, II, and III were observed at 1, 3, 7, 14, 21, and 28 d post-treatment. At the end of each observation period, the evaluation of the treatment started on live animals, including clinical evaluation and MRI then spinal cord specimens were subjected to histopathological, immunohistochemical examination, transmission electron microscopy (TEM), and QT-PCR. The timeline of the study was provided (Additional file 1: Fig. S1).

### Clinical evaluation using gait score analysis

The cats’ gait changes were measured according to the standard BBB score for the hindlimb and tail movements. BBB score ranged from 0 points with no noticeable hindlimb movement to 21 points with persistent harmonized gait, trunk stability, toe clearance, and an elevated tail. [30, 31].

### MRI

MRI was performed using a closed unit (ECHELON Smart is Hitachi’s 1.5 T Supercon MRI, Japan) under general anesthesia. The technique for spinal imaging protocol was performed in T11 to L3 (vertebral body) and included transverse T2-weighted (TR/TE 3290/99 ms) and T1-weighted (TR/TE 651/12 ms), sagittal STIR (TR/TE/TI 3310/ 61/140 ms) sequences sagittal, and dorsal T2-weighted (TR/TE 2880/111 ms) and T1-weighted (TR/TE 623/1 ms).

Then, all animals were anaesthetized with xylazine 1 mg/Kg/IM (Xyla-Ject® 2% ADWIA Co., A.R.E.) and ketamine 5% (Ketamar® 5% Sol. Amoun Co. A.R.E) I/M 10 mg/kg. After muscular relaxation, they were euthanized humanely by injecting sodium thiopental at 2.5% (Thiopental® EPICO, A.R.E) in the lethal doses of 67 mg/Kg/IV, [32] and the spinal cord (T11 to L3) was retrieved for alternative morphological assessments.

### Gross morphology of spinal cord

For further morphological assessments, the fascia and back muscles were dissected until the vertebral column's spinous process and the vertebral body was reached. Then, the spinous process was carefully severed using a bone cutter, and the spinal cord was excluded outside the vertebrae.

## Histopathology

### H&E staining

The spinal cord specimens were fixed in 10% Neutral Buffered Formalin for 24 h. The specimens were regularly processed for paraffin blocks, dehydrated in a graded sequence of ethyl alcohol, cleared in xylene, and embedded in paraffin. Sections of 4–5 μm thickness were obtained and stained by H&E [33] for light microscopy. The stained slides were examined using an Olympus BX43 microscope (Olympus, Tokyo, Japan), and the images were captured using an Olympus Dp27 digital camera (Olympus, Tokyo, Japan). Lesion score was performed for the detected histopathological alterations on a scale ranging from 0 to 3 concerning severity (0 = absent, 1 = mild, 2 = moderate and 3 = severe) for each of the following lesions: inflammation, hemorrhage, neuronal degeneration, and demyelination. The total histopathological lesion score was obtained by summation of the fore mentioned scores.

#### Immunohistochemistry

5-µm tissue sections were fixed into adhesive slides, rehydrated to water, and subjected to heat-induced epitope retrieval in a microwave for 15 min. After washing, tissue sections were incubated with primary antibodies (Anti-MBP sc-271524; Anti-GFAP sc-33673; Anti-Olig2 sc-293163; Anti-Bax sc-7480, Santa Cruz Biotechnology, Inc., Heidelberg Germany) at a dilution of 1:150 for 12 h in a refrigerator. Furthermore, sections from the SVF co-treated group were incubated with primary antibody against CD44 at a dilution of 1:1000. Then additional washing steps. Later, HRP-labeled secondary antibodies (Goat anti-mouse HRP-labeled secondary antibody, ABCam, UK) were added at a dilution of 1:1000 for two hours at room temperature. To develop the color, DAB-Substrate Kit was used. Control negative slides were performed by deleting the primary antibody. Positive immunostaining was quantified as area percentage (n = 5 slides representing 5 specimens per group, 3 microscopic fields from each slide) using cellSens dimensions (Olympus software).

#### TEM

Small, 1-mm spinal cord specimens from all groups were fixed in 3% glutaraldehyde in 0.1 M phosphate buffer for a few hours before being post-fixed in 1% osmium tetroxide for one hour. Then, 1-µm semi-thin sections were cut and stained with toluidine blue. Selective ultrathin sections were cut and stained with uranyl acetate and lead citrate. Finally, the obtained sections were analyzed via TEM et al.-Azhar University’s Regional Center for Mycology and Biotechnology (RCMP) (JEOL 1010). Furthermore, the myelin sheath thickness was assessed in at least 30 myelinated axons/samples using the ImageJ program.

#### Gene expression

Total RNA was extracted using the easy-spin Total RNA Extraction Kit (iNtRON Biotechnology DR, Cat. No.17221) as directed by the manufacturer. A Nanodrop ND-1000 spectrophotometer was used to assess the quality and quantity of RNA (Nanodrop Technologies). The cDNA was created using M-MuLV Reverse Transcriptase (NEB#M0253) as per the provided protocol. Real-time reverse transcription (RT)-PCR was used to examine the target gene expression, and mRNA levels were determined using qRT-PCR with the HERAPLUS SYBR Green qPCR kit (#: WF10308002). The primer sets are presented in Table 1. The following were the cycle conditions: 95 °C for 2 minutes, followed by 40 cycles of 95 °C for 10 seconds and 60 °C for 30 s. Each RT-PCR was conducted in triplicate [34]. The GAPDH gene was used as an internal control. The qRT-PCR results were assessed using CT, Δ CT, ΔΔ CT, and 2^− ΔΔCT^ [35].

**Table 1:** Primers sequences used for qRT-PCR

Gene symbol	Primer sequence	Accession number	Annealing temperature °C	Amplicon size bp
*Bdnf*	Forward primer: 5′-CGGTCACCGTCCTTGAAAA-3′ Reverse primer: 5′-GGATTGCACTTGGTCTCGTAGAA-3′	NM_001009828.1	60	76
*Gapdh*	Forward primer: 5′-TGGAAAGCCCATCACCATCT-3′ Reverse primer: 5′-CAACATACTCAGCACCAGCATCA-3′	NM_001009307.1	60	77
*Sdf-1*	Forward primer: 5′ACAGATGTCCTTGCCGATTC-3′ Reverse primer: 5′-CCACTTCAATTTCGGGTCAA -3′	XM_006937984.5	59	152
*Ngf*	Forward primer: 5′-GCAGGGCAGACCCGCAACAT-3′ Reverse primer: 5′-GCACCACCCGCCTCCAAGTC-3′	XM_045033321.1	60	140

#### Statistical analysis

A two-way ANOVA was applied to the data from completely random samples to examine the gait scores. The Fisher’s post hoc test was used to compare the effects of the various treatments. The significant differences between the groups and experiment days were indicated in the columns by letters. Values are presented as mean ± SEM (n = 5 cats/group). Different superscript letters indicate a significant difference at *P* ≤ 0.05. The statistical software package Origin Pro, version 2016, was used to calculate Pearson’s correlation coefficient. The gene expression analysis, immunohistochemistry, and transmission electron microscopy (TEM) results were analyzed using GraphPad Prism version 8.4.3 (686) in a one-way ANOVA, and the values are presented as mean ± SEM (n = 5 cats/group). Different superscript letters indicate a significant difference at *P* ≤ 0.05.

## Results

### Gross morphology of the spinal cord

**Fig. 1:**
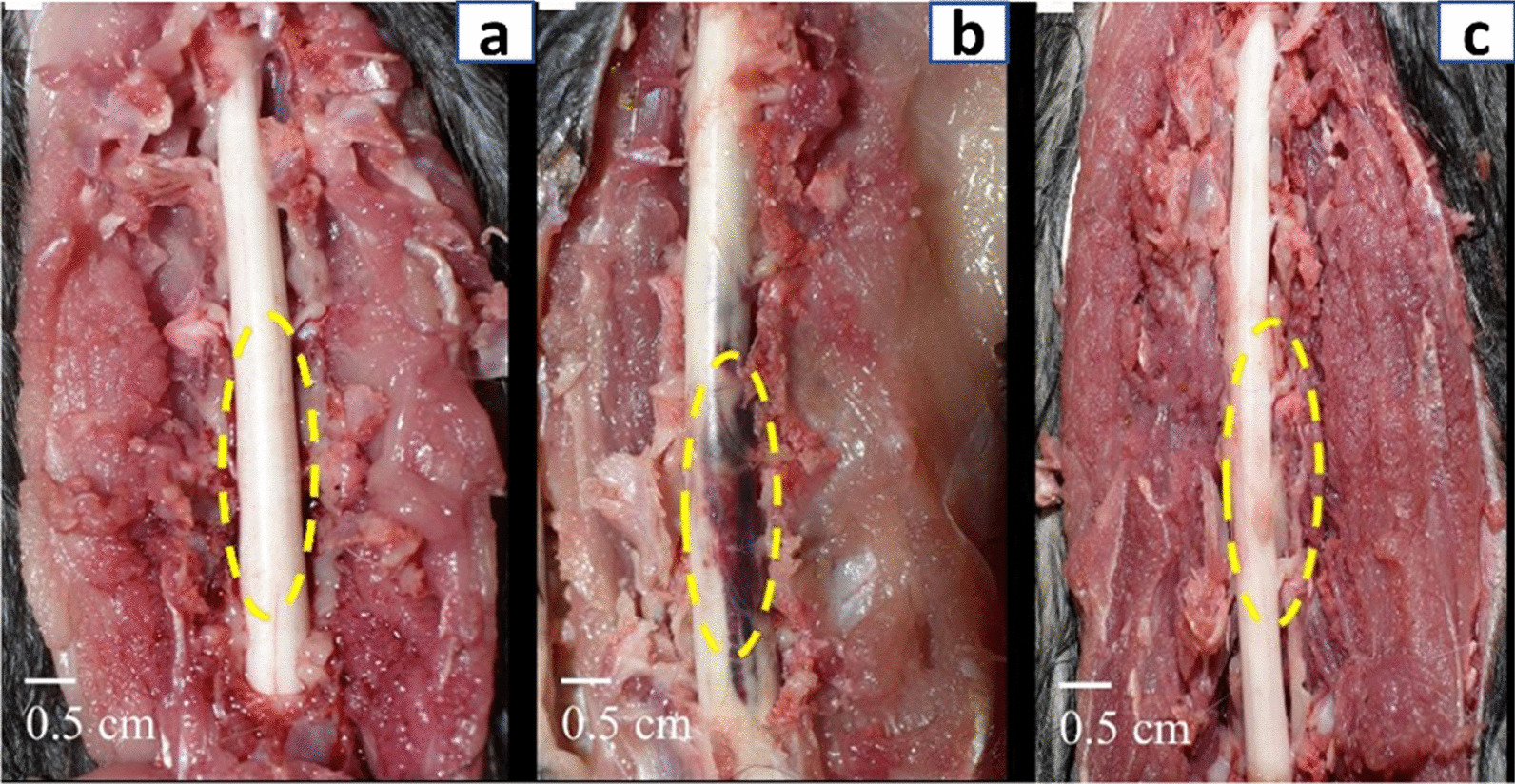
Gross morphology of the spinal cord at day 28 post-treatment **a** control negative group, **b** EB-treated group, and **c** SVF co-treated group

In the control negative group, the spinal cord segments (T11–L3) measured 6–6.5 cm. The cord appeared like a whitish-long cylindrical tube enclosed by the dura mater with no gross lesion. However, in the EB-treated group, the spinal cord was defined by the existence of a noticeable reddish-brown hemorrhagic sizable region (2–3 cm). The spinal cord of the SVF co-treated group seemed to be a whitish tube with a small localized brown lesion (< 1 cm) (Fig. 1).

### Clinical analysis post-MS induction based on the BBB score

During the study period, the functional behavior of each group of animals was evaluated by two unbiased persons using the BBB score. The BBB score of the groups was 21 before SCI.

All MS-induced cats manifested complete pelvic limb paralysis with zero or one score, one day after SCI. The gait score gradually improved and gained around three points by the end of 2 weeks. The cats suffered from urinary bladder paralysis 7 days after induction and required daily urinary bladder evacuation.

### Gait analysis after SVF treatment

Gait analysis using the BBB score revealed a significant difference between the SVF co-treated and other groups. The BBB score of the SVF co-treated group increased significantly over time to the end of 28 days. In the control negative group, the BBB score altered insignificantly between the days of the experiment, while in the EB-treated group, a significant difference between days 1, 3, and 10, but no difference beyond that, with a maximum score of five points.

**Fig. 2:**
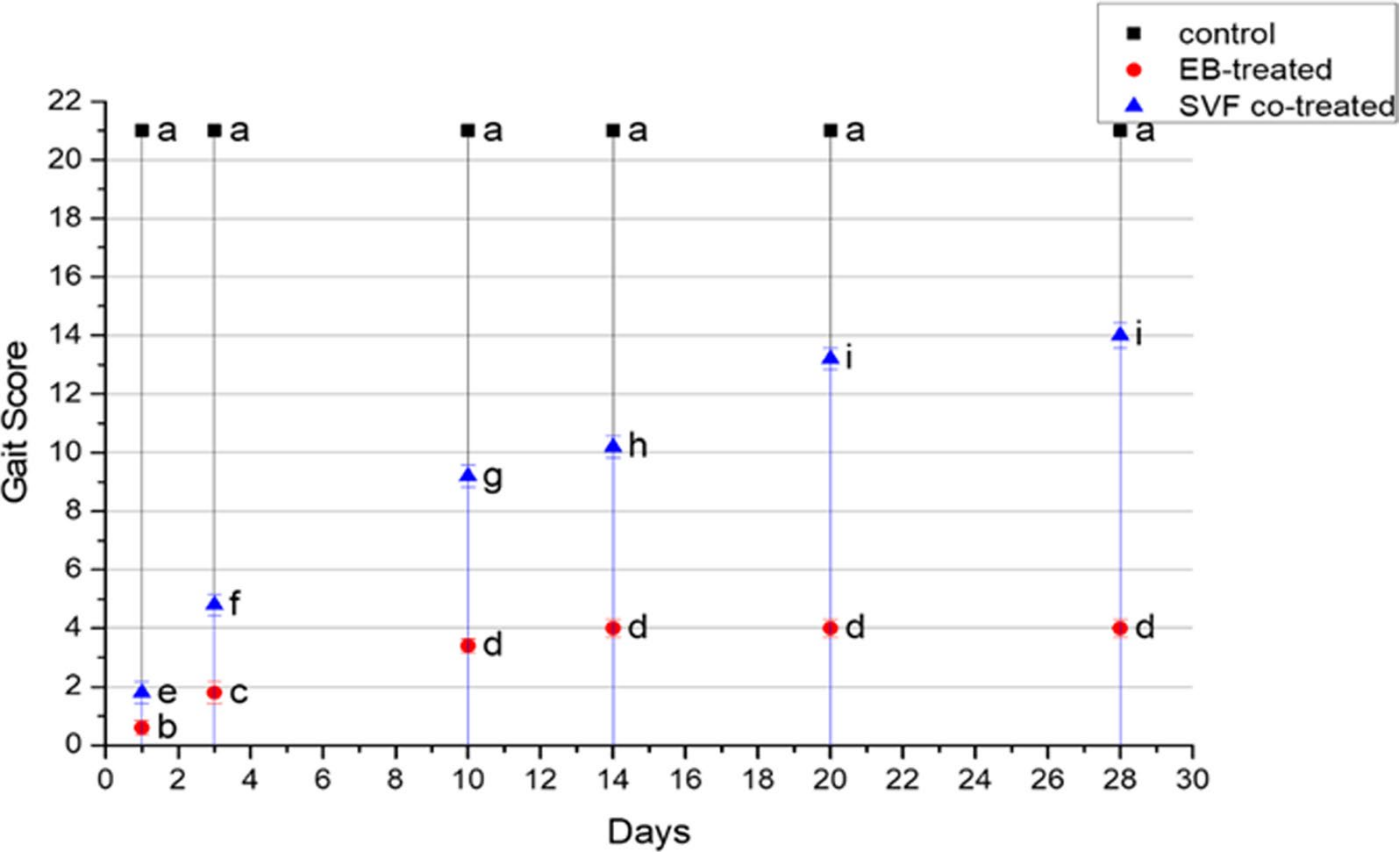
Graphical illustration of the hindlimb gait score assessment using BBB score. Values are presented as mean ± SEM (n = 5 Cats/group). Different superscript letters indicate a significant difference at *P* ≤ 0.05

In the SVF co-treated group, functional recovery of the hindlimb gradually improved following SVF transplantation. Three days after transplantation, cats had scored around five to six points. Ten days after transplantation, cats had BBB scores of approximately nine to ten points, and cats could urinate normally without assistance. Fourteen days after transplantation, cats had BBB scores ranging from 10 to 11 points. Twenty days after transplantation, cats had BBB scores ranging from 13 to 14 points. Finally, 28 days after transplantation, cats had BBB scores ranging from 14 to 15 points. There was a significant difference between the days of evaluation, as shown in Fig. 2.

### MRI analysis

**Fig. 3:**
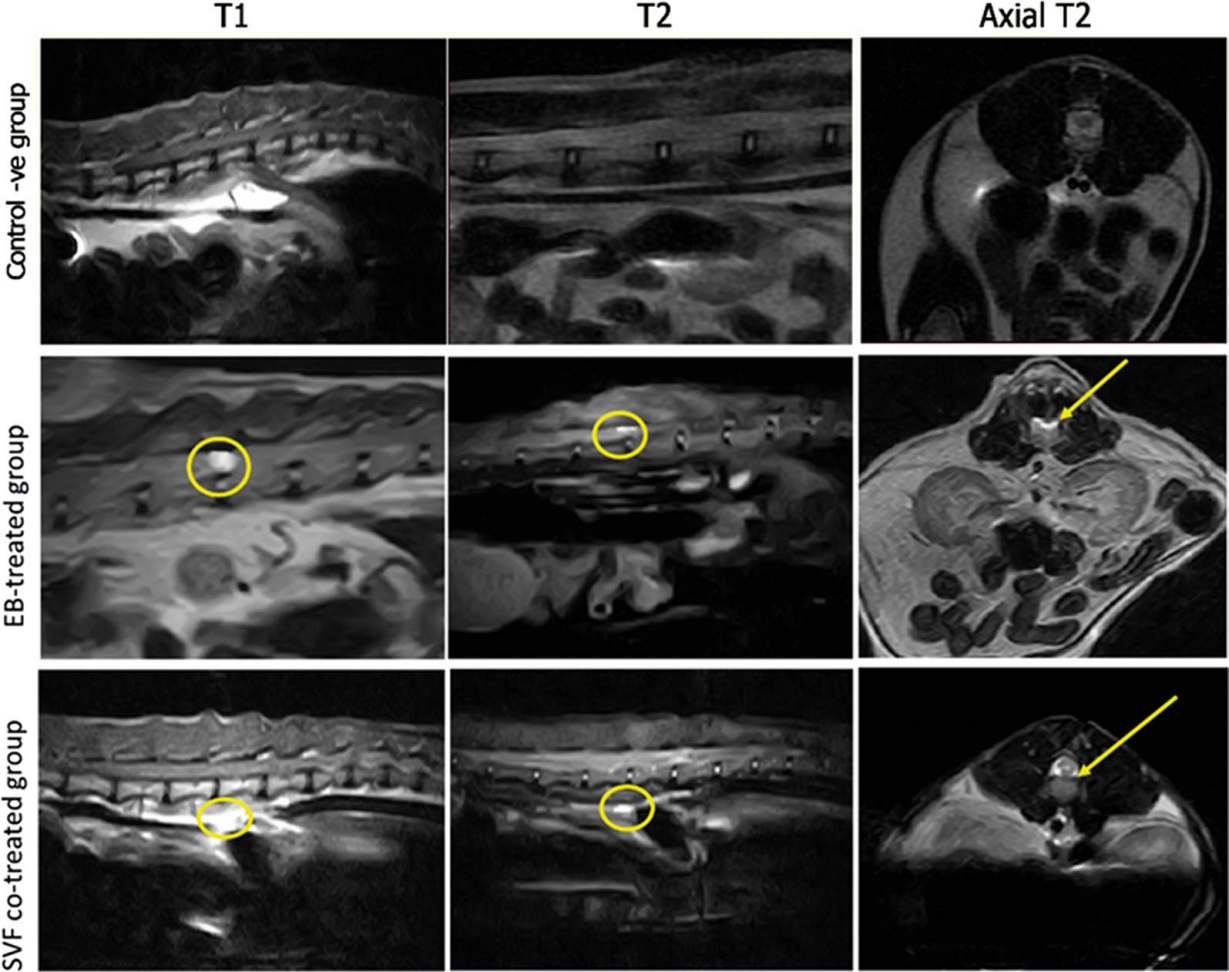
MRI of treated groups at day 28 post-treatment. Control negative group provides normal radiological images of spinal cord. EB-treated group has large hypointense lesion (circle) on sagittal T1 scan, diffuse hyperintense lesion on sagittal (circle) and axial (arrow) T2 scan, while in SVF co-treated group has small faint hypointense lesion on sagittal T1 scan, faint hyperintense lesion on sagittal and axial T2 scan, showing decreased intensity and extent of lesion (circle & arrow)

An MRI analysis was performed to indicate the degree and extent of the injury at the T13–L1 level. Cats in the EB-treated group had a diffuse, hyperintense lesion on sagittal and axial T2-weighted images and a hypointense lesion on sagittal T1-weighted images, indicating the presence of sclerotic plaque. On T2, T1 sagittal, and axial-weighted images, the SVF co-treated group had relatively small faint lesions (Fig. 3).

### Characterization of ADMSCs

#### Morphology of cells

Cells appeared rounded with few cells possessing a spindle shape under a bright-field inverted microscope at day 3 post-culture. After 10 days of the culture, the majority of cells are attached to the flask and possess a spindle shape (Additional file 1: Fig. S2).

#### Flow cytometry analysis of ADMSCS

The cells of passage three and after showed high expression for CD105 and CD73 markers (not less than 60%) and very low expression for CD34 and CD45, indicating the criteria of ADMSCS (Additional file 1: Fig. S3).

#### ADSCs differentiation

ADSC differentiation potential was investigated through their chondrogenic and adipogenic differentiation, and the cells exhibited positive staining with Safranin-O and Oil Red O, respectively (Additional file 1: Fig. S4).

### Histopathology

#### H&E staining

**Fig. 4:**
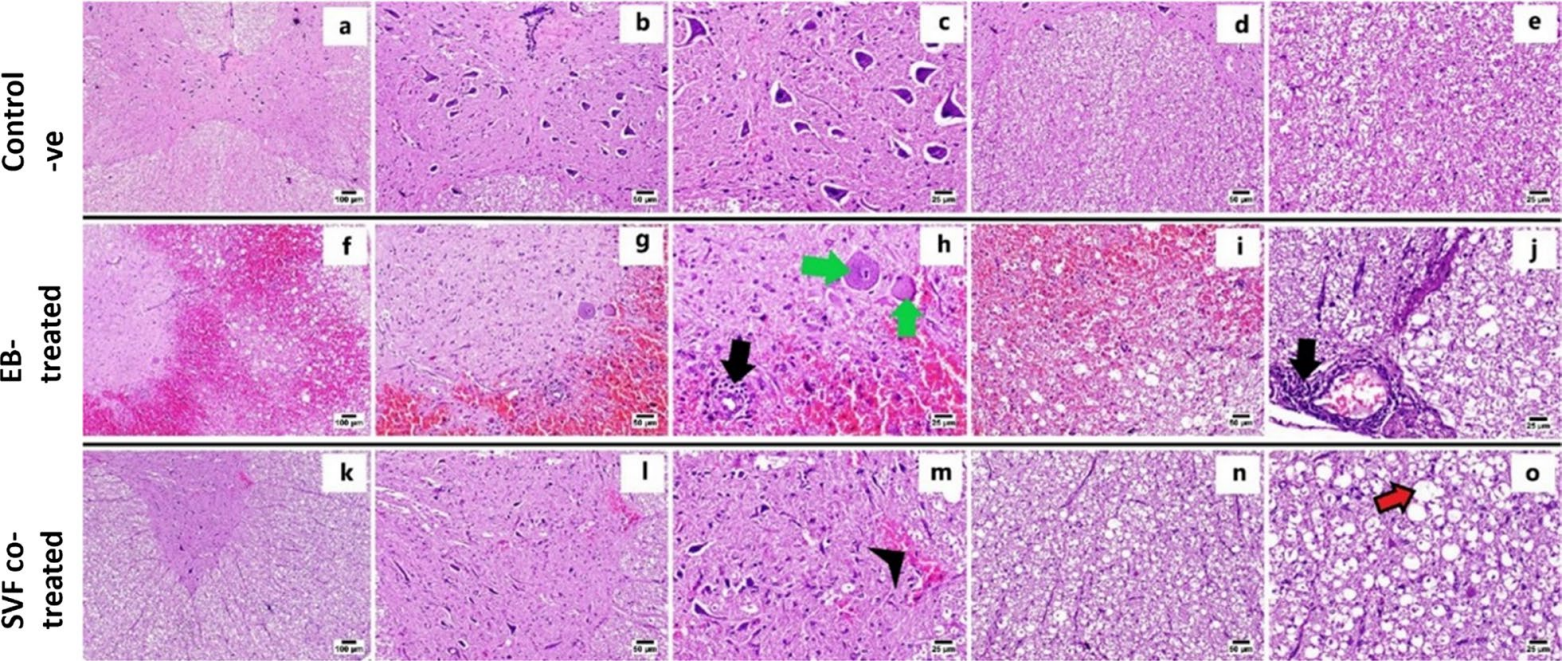
Photomicrograph of spinal cord of different experimental groups, (H&E) at day 28 post-treatment: **a**–**e** Control group showing normal histological structure of gray matter (**b**, **c**) and white matter (**d**, **e**). **f**–**j** EB-treated group showing **f** severe extensive hemorrhage in gray and white matters, **g** hemorrhage with perivascular lymphocytic cuffing in gray matter, **h** higher magnification, hemorrhage with perivascular lymphocytic cuffing (black arrow) and chromatolysis (green arrows), **i** hemorrhages in white matter, **j** congested blood vessel with perivascular lymphocytic infiltration in white matter (arrow). **k**–**o** SVF co-treated group showing, **l**, **m** apparently normal spinal cord with limited hemorrhages in gray matter (arrowhead), and **n**, **o** apparently normal nerve fibers with mild demyelination in white matter (red arrow)

Light microscopic examination of the control negative group (Fig. 4a–e) revealed the normal histological structure of both gray and white matter. Meanwhile, the EB-treated group (Fig. 4f–j) revealed several histopathological changes. Extensively diffused and severe hemorrhages were detected in white and gray matters associated with marked demyelination characterized by marked vacuolation associated with the formation of numerous digestion chambers. The gray matter suffered from diffuse gliosis, degenerated neurons, and chromatolysis. Moreover, perivascular lymphocytic cuffing was frequently noticed in the affected individuals. There was a marked improvement in the SVF co-treated group (Fig. 4k–o). The white matter showed apparently normal nerve fibers in several examined sections with minimal demyelination. Limited focal hemorrhagic areas were observed in the gray matter with apparently normal neurons (Fig. 4).

**Fig. 5:**
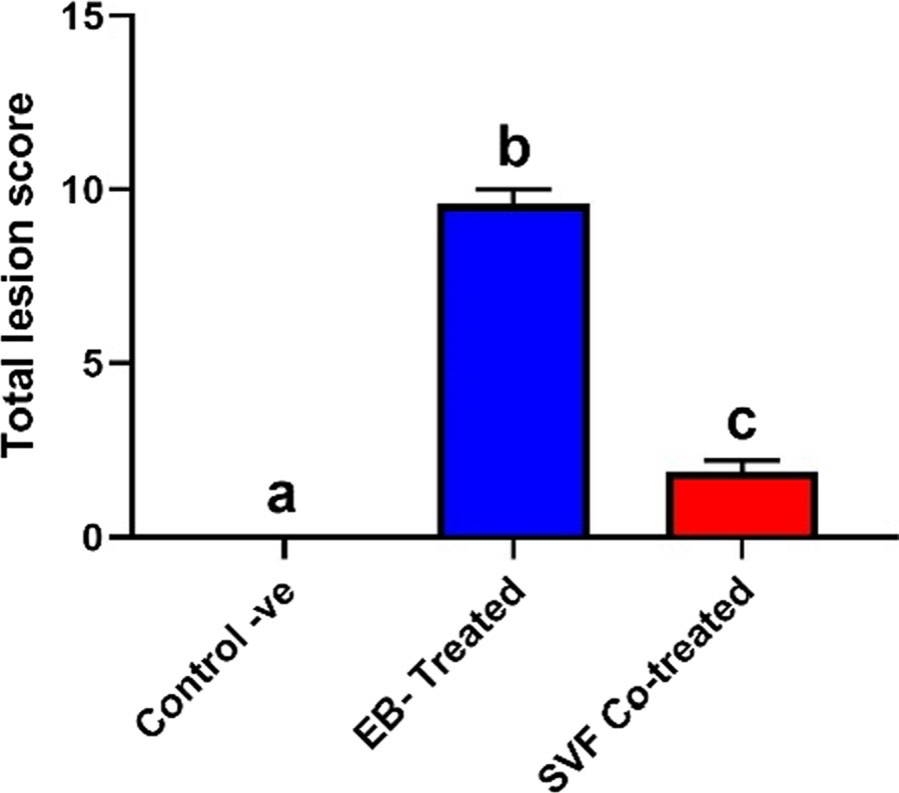
Chart presenting total histologic lesion score in different groups. Data are presented as means ± SEM. Different superscript letters indicate a significant difference at *P* ≤ 0.05

The total histologic lesion score is illustrated in Fig. 5; the SVF Co-treated group exhibited a significantly lower score in comparison with the EB-treated group.

#### Immunohistochemistry

##### Bax expression

**Fig. 6:**
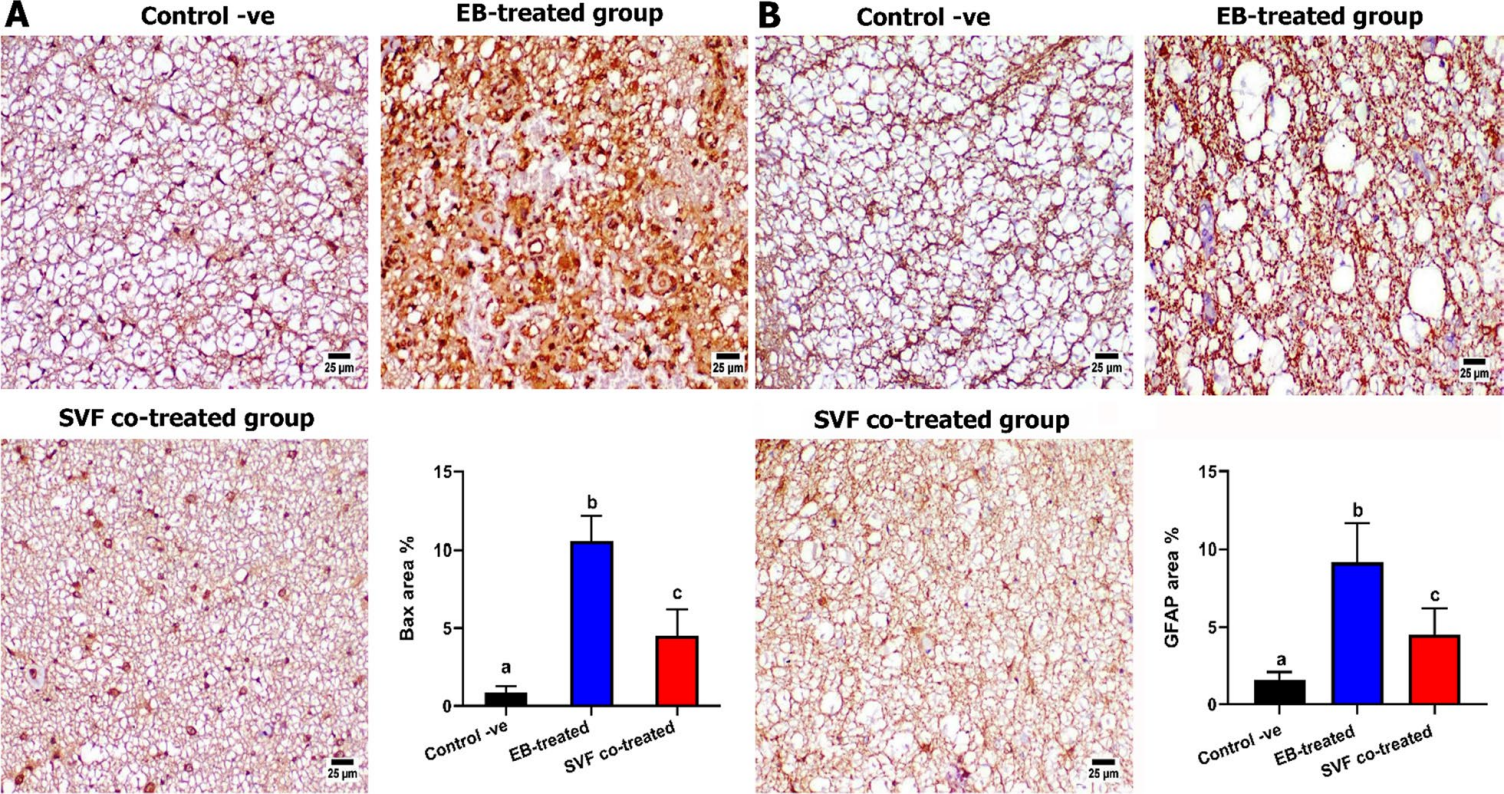
Photomicrographs of spinal cords of different groups at day 28 post-treatment showing **a** Bax expression showing a strong positive staining in the EB-treated group with a marked reduction in the SVF co-treated group. **b** GFAP expression revealing an increased positive expression in the EB-treated group with a limited expression in the SVF co-treated group. Charts presenting quantification of positive staining as area percent. Values are presented as mean ± SEM (n = 5 cats/group). Different superscript letters indicate a significant difference at *P* ≤ 0.05

A strong positive expression was detected in the EB-treated group, while a marked decrease in Bax expression was detected in the SVF co-treated group compared with the EB-treated group (Fig. 6a).

##### GFAP expression

Compared with the other experimental groups, the EB-treated group showed significantly higher levels of GFAP expression. The SVF co-treated group showed a significant reduction in GFAP-positive staining compared with the EB-treated group (Fig. 6b).

##### Olig2 expression

**Fig. 7:**
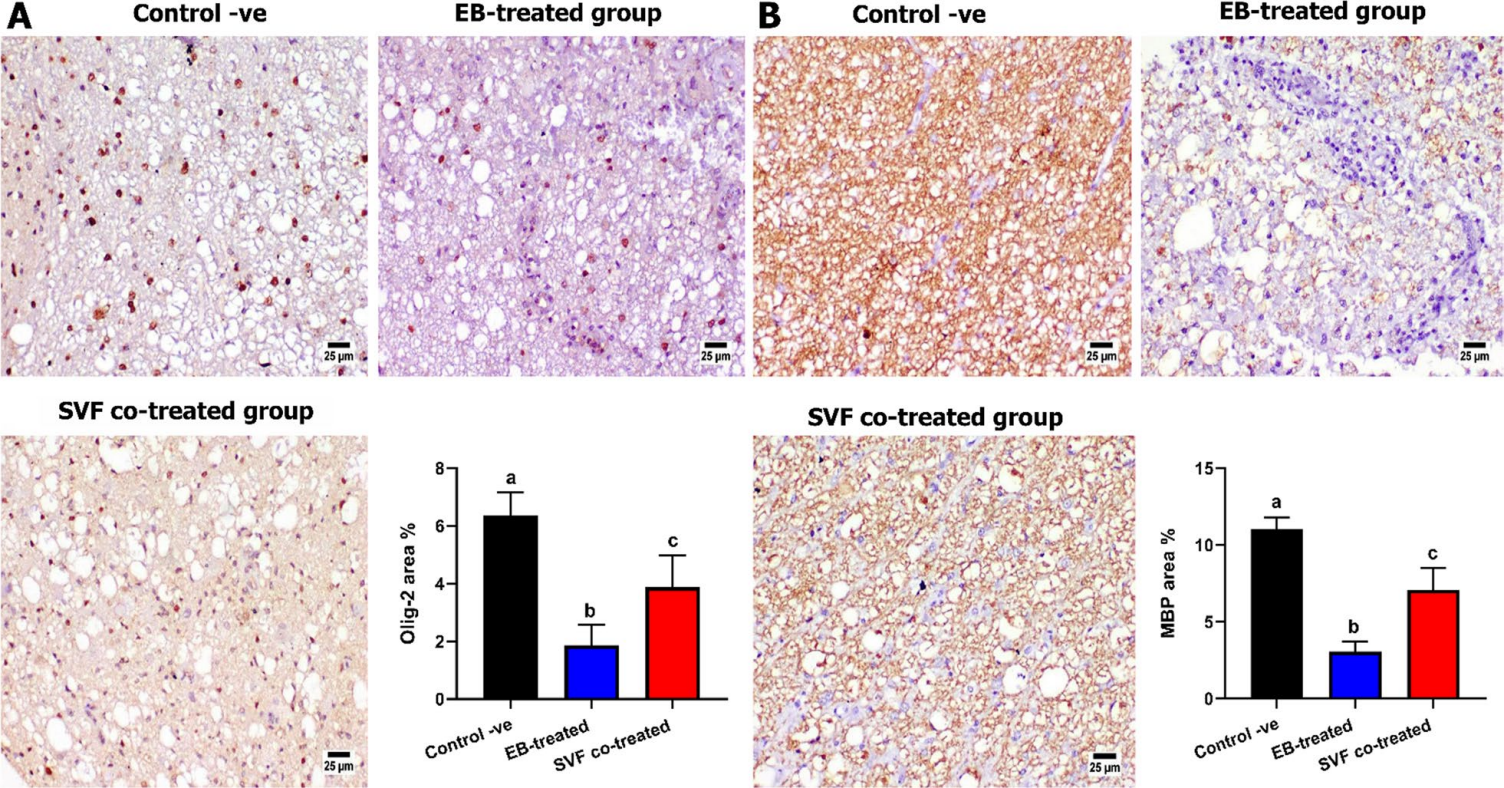
Photomicrographs of spinal cord of different groups at day 28 post-treatment showing **a** Olig2 expression demonstrating a normal expression in the control -ve group, a reduced expression in the EB-treated group with an increased expression in the SVF co-treated group. **b** MBP expression, EB-treated group exhibiting a marked reduction in positive immune expression with normal expression in the control -ve group and a marked improvement in the SVF co-treated group. Charts showing quantification of positive staining as area percent. Values are presented as mean ± SEM (n = 5 cats/group). Different superscript letters indicate a significant difference at *P* ≤ 0.05

Olig2-positive cells significantly increased in the SVF co-treated group compared with the EB-treated group which exhibited a marked reduction in Olig2-positive cells (Fig. 7a).

##### MBP expression

Normally, a higher expression of MBP was detected in the control group compared with other groups. Limited expression was observed in the EB-treated group, which showed a significant decrease compared with the other groups. Meanwhile, the SVF co-treated group exhibited a significant increase in MBP-positive staining (Fig. 7b).

#### CD44 expression

**Fig. 8:**
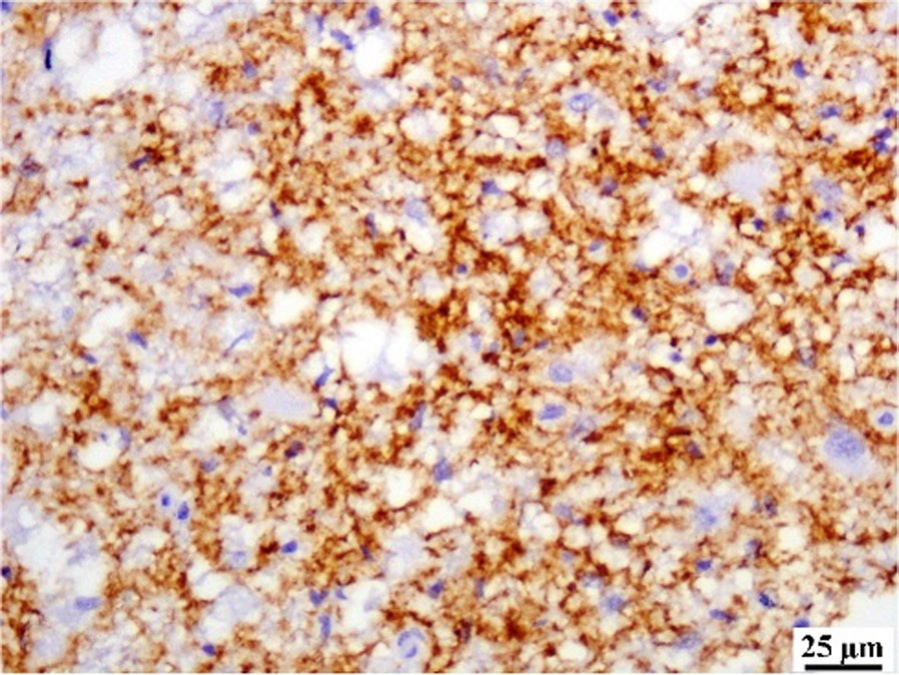
A photomicrograph of spinal cord from SVF co-treated group showing positive immune expression for CD44. (× 400; Scale bars 25 µm)

SVF co-treated group showed a strong positive immunoreactivity against CD44 as presented in Fig. 8.

#### TEM

**Fig. 9:**
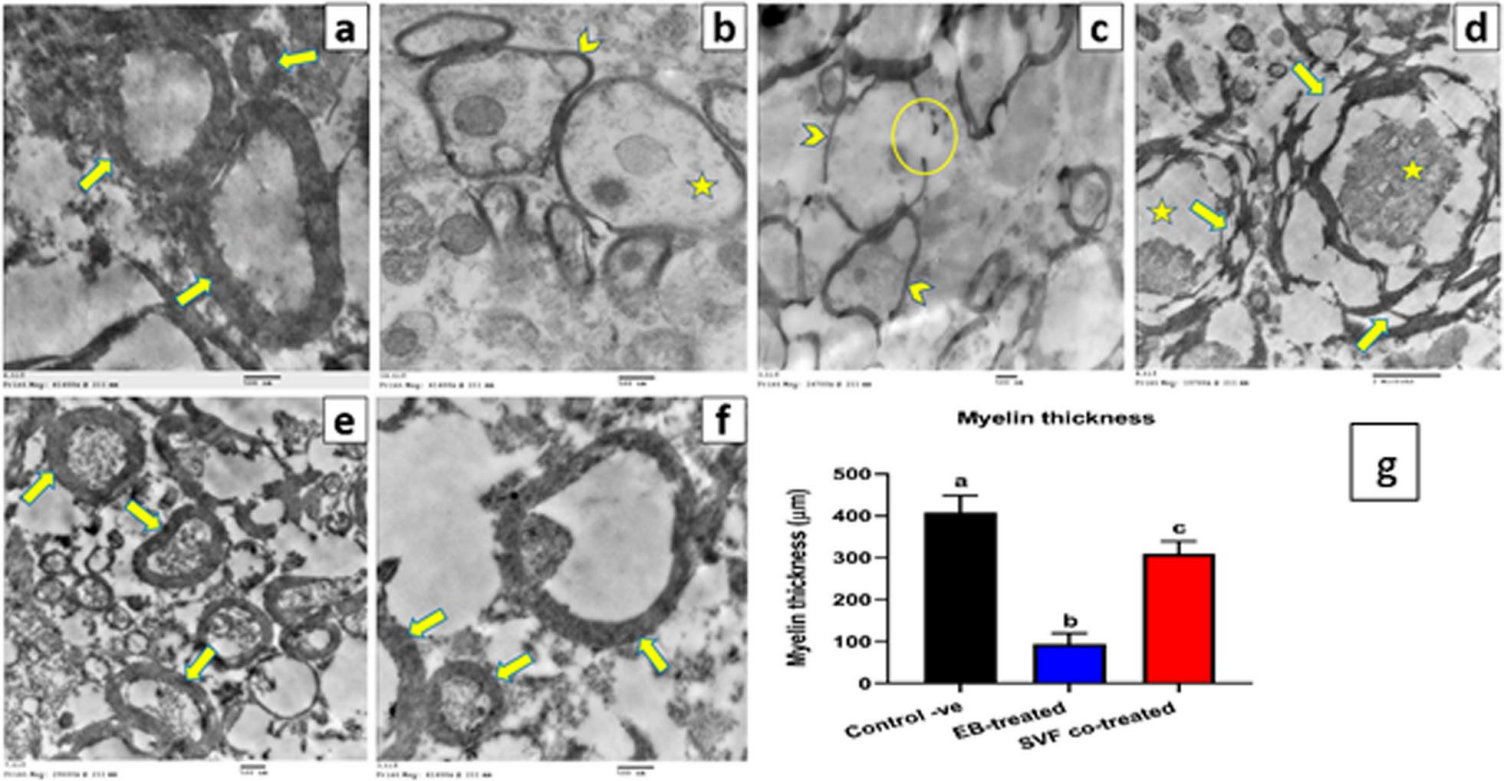
Electron micrograph shows changes in the myelin sheath of the spinal cord sections in different groups without SVF. **a** Control group with regular, electron dense, compact myelin sheaths (arrow) (20000X) **b**–**d** EB-treated group with demyelinating features including axonal swelling and degeneration (star), thinning (arrowhead), discontinuation (circle), and splitting (arrow) of the myelin sheaths (b:20000X, c:10000X, d:8000X) **e**, **f** SVF co-treated group demonstrating reconstructed structure with regular more compact, and thicker myelin sheaths (arrow) (e:12000X, f:20000x). **g** Charts presenting changes in the myelin thickness of different groups. Data are presented as means ± SEM. Groups having different letters are significantly different at *P* value < 0.05

Ultrastructural analysis displayed intact myelinated nerve fibers with normal myelin distribution and density in the control group (Fig. 9a). Conversely, the EB-treated group revealed demyelinating features of spinal cord axons, including axonal swelling, and degeneration, as well as thinning, disintegration, and splitting of the myelin sheaths (Fig. 9b–d). Conversely, reconstructed structures with more regular, compact, electron dense and thicker myelin sheaths surrounding intact axons were observed in the SVF co-treated group (Fig. 9e, f).

As presented in Fig. 9g, assessment of TEM images revealed a significant (*P* value < 0.05) decrease in the mean myelin thickness of spinal cord nerve fibers in the EB treatment group compared with the control group. Conversely, the SVF co-treated group revealed a substantial (*P* value < 0.05) increase in the mean myelin thickness compared with the EB treatment group (Fig. 9). Furthermore, there was a significant (*P* value < 0.05) difference in the myelin thickness between the control and SVF co-treated groups.

### RT-PCR gene expression

**Fig. 10:**
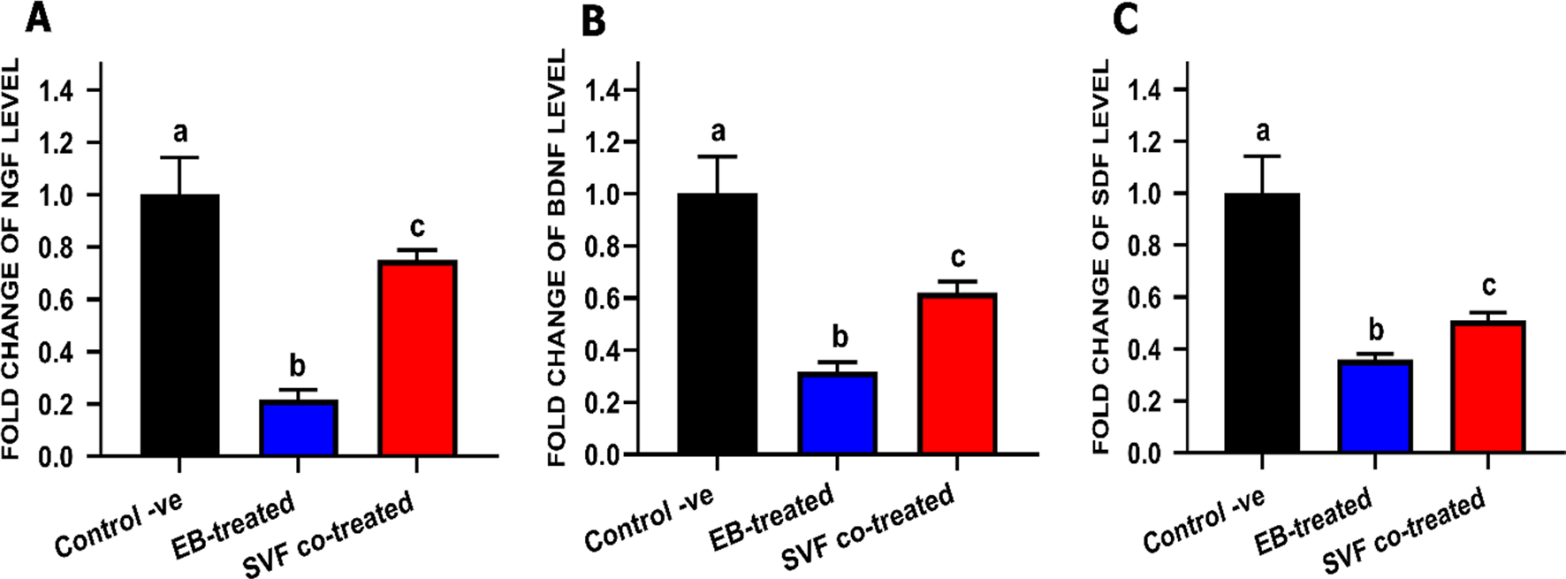
Bar chart representing the transcript levels of **a** NGF, **b** SDF, **c** BDNF In different groups at day 28 post-treatment. Values are presented as mean ± SEM (n = 5 cats/group). Data were calculated by the 2^−ΔΔCT^ method, using GAPDH as internal control, and then normalized to the control negative value which was set to be 1. Different superscript letters indicate a significant difference at *P* ≤ value 0.05

The neurotrophic factor expression was examined to evaluate the role of SVF at a molecular level. Glyceraldehyde 3-phosphate dehydrogenase (GAPDH) was used as an internal control. The EB-treated group showed significantly (*P* value < 0.05) decreased levels of *NGF*, *SDF*, and *BDNF*. The SVF co-treated group significantly (*P* value < 0.05) increased the transcript levels of the studied genes. These results confirmed the protective role of SVF against EB-induced damage (Fig. 10).

## Discussion

A number of animal models have been employed in order to better understand the pathogenesis of MS and develop new therapeutic approaches. These models include the immune-mediated model, viral-induced models, and toxin-induced demyelination models caused by cuprizone, lysolecithin, and ethidium bromide; the substances are the most frequently utilized to induce focal demyelination and destruct oligodendrocyte. The cuprizone effect was studied in rats [36, 37] and mice [38], the lysolecithin effect was reported in mice by [39] and rats [40], while the ethidium bromide effect was studied in mice model [41], rat [24, 42], dog [43], and cat [44].

The spinal cord injury model of MS in our cats is potentially improved by the intrathecal implantation of the SVF with minimal side effects. This improvement was evaluated by the BBB score, MRI, and remyelination capacity after an injury, which was not recorded in Persian cats throughout the previous studies. The other studies investigated the therapeutic effects of SVF in spinal cord injury in the mouse model [45], in the dog model [46], in the domestic ferret [47], and MS in dogs [23] and mice [48]. However, [49] declared that SVF did not regenerate growth plate injury in rats.

Our findings showed that injecting EB into the spinal cords of cats resulted macroscopically in hindlimb dysfunction, decreased proprioceptive reflexes, and urinary bladder paralysis. Microscopically there were focal demyelination areas that looked like MS attacks confirmed by [50]. EB effects were reported in animal models like a rat by [24, 42], in mice by [41], and dogs by [23]. This helped us evaluate a new treatment and other reparative techniques.

For spinal and Intra articular injections of SVF less than one billion cells are considered a safe dose while for intravenous injections less than 10 billion SVF cells in 250 cc of normal saline are considered to be safe reported by [51]. Our results showed that injection of 10 × 10^6^ nucleated cells intrathecal directly into the CSF was a safe cell number with no side effects which is in line with [23, 52].

In our treatment, we depended on SVF, so we first activated it using low laser irradiation, [53, 54] mentioned that the optimal use of laser irradiation at 5 J/cm2, 5.5 mW/cm2 power density, and 635 nm wavelength which enhanced the SVF activation, cell survival, proliferation, vascularization potential, and expression of β1-integrin with no side effects in line with [23, 55, 56].

The results of this study showed that a single injection of SVF improved the hindlimb locomotion, decrease lesion severity, considerably increase the gene expression of neurotrophic factors, and improved remyelination capability which was verified in MS by [23], osteoarthritis by [52] on the other hand [54] reported that multiple injections of SVF in treatment of diabetes mellitus more efficient than single injection and also [57] mentioned that twofold injection of bone marrow pancreatic progenitor cells could treat diabetes mellitus.

Also, we confirmed through flow cytometry analysis that the CD105 and CD73 markers were highly expressed in ADMSCs, while the CD45 expression was relatively low, in line with previous studies [28, 58].

Our best method for injecting SVF was intrathecal through the foramen magnum approach, which increased the cell capacity to reach the affected areas in the spinal cord by circulating in the cerebrospinal fluid directly. It is considered a safe and effective treatment method for MS and coincides with [59, 60], and enhanced limb movement, as asserted by [61]. Conversely, [62] reported that the intrathecal approach for treating MS has little clinical improvement and no radiological change. Also, [63] reported that SVF had a great inflammatory response in goats for treating intervertebral disks.

Our assignment in each group began by using BBB scores, and the outcome in the EB-treated group was unsatisfactory. All cats had low hindlimb function ratings throughout the trial period. From the beginning through the end of the trial, the cat’s hindlimbs were paralyzed and swept when they tried moving. These findings matched those previously published by [64, 65], while the SVF co-treated group revealed a greater improvement in hindlimb locomotive function than the control group. Improvement started 10 days after receiving treatment, and cats gained the ability to urinate, and the BBB score reached 15 points at the end of the 28th day, which was agreed with [7, 23].

The second evaluation was the MRI, which showed in the EB-treated group, a diffuse hyperintense sclerotic plaque in the T2-weighted and axial scan and hypointense sclerotic plaque in the T1-weighted scan. The SVF co-treated group had a decrease in lesion intensity on the T1-weighted, T2-weighted, and axial scans as mentioned by [66] in the dog with spinal cord injury and [67]. This is unlike the study mentioned by [62], which found no improvement in MRI lesions after stem cell injection.

While our macroscopical assessment showed that the spinal cord in the EB-treated group was defined by a large clear hemorrhagic region. In contrast, the spinal cord in the SVF co-treated group appeared to be a whitish tube with a small localized brown lesion, these findings are in agreement with [68], who declared that there is a considerable variation in the morphology of the spinal cord in dogs after injury, while [1] in the dog reported that the morphology of the spinal cord did not change after the initial insult.

Our histopathological assessment showed that the EB-treated group was characterized by pathological hallmarks in both white and gray matter structures, such as significant demyelination, severe hemorrhage, axonal degeneration, and fragmentation of Nissl substance accompanied by the proliferation of glial cells. In contrast, the SVF co-treated group revealed a marked improvement in the histological architecture with minor demyelination in the white matter. These findings are similar to those of previous studies [23, 69, 70]. The demyelinating damage induced by EB and enhanced remyelination by SVF were verified using TEM analysis and Olig2/MBP immunohistochemistry. Ultrastructural analysis displayed axonal swelling and degeneration, as well as disintegration, thinning, and splitting of the myelin sheaths in the EB-treated group compared with that of the control group. These findings agreed with [23]. In contrast, the SVF co-treated group exhibited nearly normal axons with more intact and thicker myelin sheaths. This result is consistent with [23] and [71]. Previous studies demonstrated the remyelination potency, thickness of myelin sheaths, and percentage of myelinated nerve fibers that can be enhanced by ASCs (AMSCs) transplantation in the neural tissue of the cuprizone model of MS [72, 73].

Olig2 and MBP were highly associated with oligodendrocyte regeneration and maintained the myelin structure, as revealed in this study, by a significant expression in the SVF co-treated group than in the EB-treated group, as reported by [23, 74, 75]. This might be due to SVF cells differentiation into oligodendrocyte progenitors. These findings are like those that demonstrated the successful trans-differentiation of ADMSCs into neural lineages, specifically Olig2-expressing cells [76, 77].

In this work, the immunohistochemistry investigation of the examined sections showed that the GFAP expression was reduced in the SVF co-treated group, resulting in a smaller glial scar and minimal astrocyte reaction compared with a large glial scar and severe reactivity in the EB-treated group. This reveals that the SVF could inhibit astrocyte proliferation because of the EB injection, as previously mentioned by [23, 78].

Previous studies have shown that the SVF secretome contains many growth factors that can stimulate cell proliferation and prevent the expression of apoptotic factors, such as Bax; this finding was reported by [79, 80]. Our study recorded a significantly higher expression of Bax in the EB-treated group than in the control and SVF co-treated groups, which agrees with [23] in MS and [80] in denervated muscle.

The effects of SVF administration during the course of the disease were also evaluated by assessing the transcript levels of BDNF, NGF, and SDF-1. BDNF is widely disseminated in the central nervous system. It promotes neural growth and development during growth and plays an important role in regulating synaptic transmission and plasticity [81]. BDNF is involved in activity-dependent plasticity processes [82], such as long-term potentiation, learning, and memory. Our results showed BDNF upregulation in the SVF-treated rats. As the disease progresses, BDNF levels in the brains of AD and MS patients decrease [83]. Moreover, the increased BDNF level in serum correlates with improved cognitive function [84]. BDNF promotes platelet activation, aggregation, and secretion by activating shortened forms of the TrkB receptor. [85].

Treatment with SVF temporarily increases the severity of the disease. However, the mice treated with SVF cells showed improvement and mild disease severity [86]. These effects may be mediated by increased neurotrophic factors, such as NGF and BDNF. The MS patients injected with the SVF showed stability or improvement [87].

Besides their immunomodulatory capacity, SVF can produce agents that potentially stimulate cell proliferation, promote angiogenesis, and inhibit apoptosis. They include stromal cell-derived factor (SDF-1α) [88]. Furthermore, the SVF can also promote NGF production, and these factors are essential for regulating wound healing and tissue repair [89].

NGF is vital for the neurons during the differentiation of the nervous system. Significant increases in NGF synthesis in inflammatory tissues in patients with an inflammatory disease have been reported [90]. Changes in NGF synthesis have significant effects on neurophysiology, and they can also affect immune cell activity. NGF is an important molecule in the complex network of bidirectional signals between the nervous and immune systems. Initially, elevated NGF levels were seen in patients with MS cerebrospinal fluid, and increased NGF levels closely follow disease progression. In contrast to our results, the synovial fluid in patients with rheumatoid arthritis is also characterized by elevated NGF levels. Its upregulation in the inflamed synovial fluid has been reported [91].

Based on our results, SVF can improve the MS cat model through different mechanisms. Firstly, the ability of MSCs to increase migration of oligodendrocyte progenitors and differentiation into oligodendrocytes that were evidenced by a strong positive immunoreactivity against CD44 antibodies in the SVF co-treated sections. CD44 is a cell surface receptor that plays a key role in mediating cell migration and consider one of MSCs important markers. [92] recorded that MSCs increase the migration of oligodendrocyte progenitors from surrounding niches by various trophic signals from the grafted MSCs. [93] provide evidence on the direct differentiation of MSCs into oligodendrocytes by transplantation of GFP-labeled BMSCs into EAE mice model. Secondly, MSCs can increase differentiated oligodendrocyte progenitors into mature oligodendrocyte and myelin production as observed by increased immune expression of Olig2 and MPB. This result comes in accordance with [94]. Thirdly, the potent immunomodulatory, anti-apoptotic, and anti-inflammatory effect of SVF in addition to the secretion of neurotrophic factors that stimulate cell proliferation, promote angiogenesis, inhibit apoptosis, and regulate tissue repair.

## Conclusion

Our study demonstrated that the transplantation of SVF intrathecally could alleviate the adverse effects of ethidium bromide-induced demyelination in a cat model of MS. The therapeutic effects of SVF improve the motor function of the hindlimb, ameliorate the lesion severity, alleviate the induced histopathological alterations, enhance the remyelination capacity, significantly increase the gene expression of neurotrophic factors and the immunoreactivity of olig2 and MBP, and reduce Bax, and GFPA immunoreactivity. To date, this is the first investigation elucidating the global effects of SVF treatment in the cat MS model.

### Recommendations

The fast and excellent response, besides easily application of SVF injections in acute cases, gave attention to becoming a routine application in spinal cord injuries like accidents, MS, etc.

Further studies are required to evaluate the long-term and multiple dose effects of SVF in the treatment of spinal cord injuries.

## Supplementary Information

**Additional file 1.** Supplementary figures.

## Data Availability

All data collected or analyzed during this study are included in this published paper.

## Abbreviations

MS: Multiple sclerosis
SVF: Stromal vascular fraction
EB: Ethidium bromide
BBB: Basso, Beattie, and Bresnahan
NGF: Nerve growth factor
MBA: Myelin basic protein
GFAP: Glial fibrillary acidic protein
Olig 2: Oligodendrocyte transcription factor
AD: Alzheimer's disease
BDNF: Brain-derived neurotrophic factor
(FACS): Fluorescence-activated cell sorting
BSA: Bovine serum albumin
DMEM: Gibco Dulbecco's Modified Eagle Medium
EDTA: Ethylenediaminetetraacetic acid
DAB: Diaminobenzidine
S/C: Subcutaneous
HRP: Horseradish peroxidase
TrkB: Tropomyosin receptor kinase B

## References

[1] Jung DI, Ha J, Kang BT, Kim JW, Quan FS, Lee JH (2009). A comparison of autologous and allogenic bone marrow-derived mesenchymal stem cell transplantation in canine spinal cord injury. J Neurol Sci.

[2] Penha EM, Aguiar PHP, Barrouin-Melo SM, de Lima RS, da Silveira ACC, Otelo ARS (2012). Clinical neurofunctional rehabilitation of a cat with spinal cord injury after hemilaminectomy and autologous stem cell transplantation. Int J Stem Cells.

[3] Martino G, Adorini L, Rieckmann P, Hillert J, Kallmann B, Comi G (2002). Inflammation in multiple sclerosis: The good, the bad, and the complex. Lancet Neurol.

[4] Pluchino S, Furlan R, Martino G (2004). Cell-based remyelinating therapies in multiple sclerosis: evidence from experimental studies. Curr Opin Neurol.

[5] Torre-Fuentes L, Moreno-Jiménez L, Pytel V, Matías-Guiu JA, Gómez-Pinedo U, Matías-Guiu J (2020). Experimental models of demyelination and remyelination. Neurologia.

[6] Lassmann H, Bradl M (2017). Multiple sclerosis: Experimental models and reality. Acta Neuropathol.

[7] Uccelli A, Laroni A, Freedman MS (2013). Mesenchymal stem cells as treatment for MS—progress to date. Mult Scler J.

[8] Shehadi J, Elzein SM, Beery P, Spalding MC, Pershing M (2021). Combined administration of platelet-rich plasma and autologous bone marrow aspirate concentrate for spinal cord injury: A descriptive case series. Neural Regen Res.

[9] Nishida H, Nakayama M, Tanaka H, Kitamura M, Hatoya S, Sugiura K (2011). Evaluation of transplantation of autologous bone marrow stromal cells into the cerebrospinal fluid for treatment of chronic spinal cord injury in dogs. Am J Vet Res.

[10] Sarmento CAP, Rodrigues MN, Bocabello RZ, Mess AM, Miglino MA (2014). Pilot study: Bone marrow stem cells as a treatment for dogs with chronic spinal cord injury. Regen Med Res.

[11] Deng YB, Liu XG, Liu ZG, Liu XL, Liu Y, Zhou GQ (2006). Implantation of BM mesenchymal stem cells into injured spinal cord elicits de novo neurogenesis and functional recovery: evidence from a study in rhesus monkeys. Cytotherapy.

[12] Pritchard CD, Slotkin JR, Yu D, Dai H, Lawrence MS, Bronson RT (2010). Establishing a model spinal cord injury in the African green monkey for the preclinical evaluation of biodegradable polymer scaffolds seeded with human neural stem cells. J Neurosci Methods.

[13] Mcdonald JW, Liu XZ, Qu Y, Liu S, Mickey SK, Turetsky D (1999). Transplanted embryonic stem cells survive, differentiate and promote recovery in injured rat spinal cord. Nat Med.

[14] Yano S, Kuroda S, Lee JB, Shichinohe H, Seki T, Ikeda J (2005). In vivo fluorescence tracking of bone marrow stromal cells transplanted into a pneumatic injury model of rat spinal cord. J Neurotrauma.

[15] Fonseca AFB, Scheffer JP, Giraldi-Guimarães A, Coelho BP, Medina RM, Oliveira ALA (2017). Comparison among bone marrow mesenchymal stem and mononuclear cells to promote functional recovery after spinal cord injury in rabbits. Acta Cir Bras.

[16] Yang C, Wang G, Ma F, Yu B, Chen F, Yang J (2018). Repeated injections of human umbilical cord blood-derived mesenchymal stem cells significantly promotes functional recovery in rabbits with spinal cord injury of two noncontinuous segments. Stem Cell Res Ther.

[17] Ghasemi N, Razavi S, Mardani M, Esfandiari E, Salehi H, Zarkesh Esfahani SH (2014). Transplantation of human adipose-derived stem cells enhances remyelination in lysolecithin-induced focal demyelination of rat spinal cord. Mol Biotechnol.

[18] Liau LL, Looi QH, Chia WC, Subramaniam T, Ng MH, Law JX (2020). Treatment of spinal cord injury with mesenchymal stem cells. Cell Biosci.

[19] Johnson LDV, Pickard MR, Johnson WEB (2021). The comparative effects of mesenchymal stem cell transplantation therapy for spinal cord injury in humans and animal models: A systematic review and meta-analysis. Biology.

[20] Bowles AC, Strong AL, Wise RM, Thomas RC, Gerstein BY, Lipschutz RS (2015). 34. Stromal vascular fraction as a novel stem cell-based therapy for a mouse model of multiple sclerosis. Mol Ther.

[21] Nguyen A, Guo J, Banyard DA, Fadavi D, Toranto JD, Wirth GA (2015). Stromal vascular fraction: a regenerative reality? Part 1: current concepts and review of the literature. J Plast Reconstr Aesthet Surg.

[22] Chow L, McGrath S, de Arruda SC, Whalen LR, Packer R (2020). Generation of neural progenitor cells from canine induced pluripotent stem cells and preliminary safety test in dogs with spontaneous spinal cord injuries. Front Vet Sci.

[23] Abdallah AN, Shamaa AA, El-Tookhy OS (2019). Evaluation of treatment of experimentally induced canine model of multiple sclerosis using laser activated non-expanded adipose derived stem cells. Res Vet Sci.

[24] Riet-Correa G, Fernandes CG, Pereira LAV, Graça DL (2002). Ethidium bromide-induced demyelination of the sciatic nerve of adult Wistar rats. Braz J Med Biol Res.

[25] Blakemore WF (1982). Ethidium bromide induced demyelination in the spinal cord of the cat. Neuropathol ApplNeurobiol.

[26] Hayashi S, Yagi R, Taniguchi S, Uji M, Urano H, Yoshida S (2021). A novel method for processing adipose-derived stromal stem cells using a closed cell washing concentration device with a hollow fiber membrane module. Biomed Microdevices.

[27] Sekiya I, Larson BL, Smith JR, Pochampally R, Cui JG, Prockop DJ (2002). Center expansion of human adult stem cells from bone marrow stroma: conditions that maximize the yields of early progenitors and evaluate their quality. Stem Cells.

[28] Meligy FY, Abo Elgheed AT, Alghareeb SM (2019). Therapeutic effect of adipose-derived mesenchymal stem cells on Cisplatin induced testicular damage in adult male albino rat. Ultrastruct Pathol.

[29] Jankowski M, Dompe C, Sibiak R, Wasiatycz G, Mozdziak P, Jaskowski JM (2020). In vitro cultures of adipose-derived stem cells. Cells.

[30] Basso DM, Beattie MS, Bresnahan JC (1995). A sensitive and reliable locomotor rating scale for open field testing in rats. J of Neurotrauma.

[31] Farid MF, Abouelela YS, Rizk H (2021). Stem cell treatment trials of spinal cord injuries in animals. Auton Neurosci.

[32] Vassallo S (2008). Thiopental in lethal injection. React Wkly.

[33] Bancroft JD, Gamble M (2008). Theory and practice of histological techniques.

[34] Hassan N, Mostafa I, Elhady MA, Ibrahim MA, Amer H (2022). Effects of probiotic feed additives (biosol and Zemos) on growth and related genes in broiler chickens. Ital J Anim Sci.

[35] Livak KJ, Schmittgen TD (2001). Analysis of relative gene expression data using real-time quantitative PCR and the 2-ΔΔCT method. Methods.

[36] Oakden W, Bock NA, Al-Ebraheem A, Farquharson MJ, Stanisz GJ (2017). Early regional cuprizone-induced demyelination in a rat model revealed with MRI. NMR Biomed.

[37] Silvestroff L, Bartucci S, Pasquini J, Franco P (2012). Cuprizone-induced demyelination in the rat cerebral cortex and thyroid hormone effects on cortical remyelination. Exp neurol.

[38] Oveland E, Ahmad I, Lereim RR, Kroksveen AC, Barsnes H, Guldbrandsen A (2021). Cuprizone and EAE mouse frontal cortex proteomics revealed proteins altered in multiple sclerosis. Sci rep.

[39] Keough MB, Jensen SK, Yong VW (2015). Experimental demyelination and remyelination of murine spinal cord by focal injection of lysolecithin. JoVE.

[40] Zhang M, Hugon G, Bouillot C, Bolbos R, Langlois JB, Billard T (2019). Evaluation of myelin radiotracers in the lysolecithin rat model of focal demyelination: beware of pitfalls!. Contrast Media Mol Imaging.

[41] Kuypers NJ, James KT, Enzmann GU, Magnuson DS, Whittemore SR (2013). Functional consequences of ethidium bromide demyelination of the mouse ventral spinal cord. Exp neurol.

[42] Goudarzvand M, Choopani S, Shams A, Javan M, Khodaii Z, Ghamsari F (2016). Focal injection of ethidium bromide as a simple model to study cognitive deficit and its improvement. Basic Clin Neurosci.

[43] Abdallah AN, Shamaa AA (2020). Ethidium bromide induced demyelination of the central nervous system in a dog model of secondary progressive multiple sclerosis. J Curr Vet Res.

[44] Farid MF, Abouelela YS, Yasin NA, Mousa MR, Ibrahim MA, Prince A (2022). A novel cell-free intrathecal approach with PRP for the treatment of spinal cord multiple sclerosis in cats. Inflamm Regener.

[45] Lam HT, Tran MN, Bui KA, Le TT, Bui KH, Phan NK (2016). Adipose tissue derived stromal vascular fraction transplantation can recover spinal cord injury in mice. Prog Stem Cell.

[46] Raghuvanshi P, Maiti S, Tiwari S, Yadav D, Sharda R, Dewangan R (2021). Rehabilitation of spinal cord injury with autogenous stromal vascular fraction in dogs. Int Phys Med Rehabil J.

[47] Przekora A, Juszkiewicz L (2020). The effect of autologous adipose tissue-derived mesenchymal stem cells’ therapy in the treatment of chronic posttraumatic spinal cord injury in a domestic ferret patient. Cell Transplant.

[48] Bowles AC, Wise RM, Gerstein BY, Thomas RC, Ogelman R, Manayan RC (2017). Adipose stromal vascular fraction-mediated improvements at late-stage disease in a murine model of multiple Sclerosis. Stem Cells.

[49] Sananta P, Oka RI, Dradjat RS, Suroto H, Mustamsir E, Kalsum U (2020). Adipose-derived stromal vascular fraction prevent bone bridge formation on growth plate injury in rat (in vivo studies) an experimental research. Ann Med Surg.

[50] Armstrong RC (2007). Growth factor regulation of remyleination: Behind the growing interest in endogenous cell repair of the CNS. Future Neurol.

[51] Karina K, Rosliana I, Rosadi I, Schwartz R, Sobariah S, Afini I (2020). Safety of technique and procedure of stromal vascular fraction therapy: from liposuction to cell administration. Scientifica.

[52] Frisbie DD, Kisiday JD, Kawcak CE, Werpy NM, McIlwraith CW (2009). Evaluation of adipose-derived stromal vascular fraction or bone marrow-derived mesenchymal stem cells for treatment of osteoarthritis. J Orthop Res.

[53] Mvula B, Mathope T, Moore T, Abrahamse H (2008). The effect of low level laser irradiation on adult human adipose derived stem cells. Lasers med sci.

[54] Sayed SY, Salem SI, Abdallah AN, Khalil GM, Mohammed FF (2019). Clinicopathological studies on the use of laser-activated adipose-derived stromal vascular fraction in treatment of streptozotocin-induced diabetes in rats. Comp Clin Pathol.

[55] Abdallah AN, Shamaa AA, El-Tookhy OS, Abd El-Mottaleb EM (2016). Evaluation of low level laser-activated stromal vascular fraction as a single procedure for treatment of experimental chondral defects. Asian J Anim Sci.

[56] Priglinger E, Maier J, Chaudary S, Lindner C, Wurzer C, Rieger S (2018). Photobiomodulation of freshly isolated human adipose tissue-derived stromal vascular fraction cells by pulsed light-emitting diodes for direct clinical application. J Tissue Eng Regen Med.

[57] Rizk H, Tohamy AF, Sayed WM, Prince A (2018). Ameliorative effects of bone marrow derived pancreatic progenitor cells on hyperglycemia and oxidative stress in diabetic rats. Acta Histochem.

[58] Meligy FY, Shigemura K, Behnsawy HM, Fujisawa M, Kawabata M, Shirakawa T (2012). The efficiency of in vitro isolation and myogenic differentiation of MSCs derived from adipose connective tissue, bone marrow, and skeletal muscle tissue. Vitr Cell Dev Biol - Anim.

[59] Karussis D, Karageorgiou C, Vaknin-Dembinsky A, Gowda-Kurkalli B, Gomori JM, Kassis I (2010). Safety and immunological effects of mesenchymal stem cell transplantation in patients with multiple sclerosis and amyotrophic lateral sclerosis. Arch Neurol.

[60] Sahraian MA, Bonab MM, Baghbanian SM, Owji M, Moghadasi AN (2019). Therapeutic use of intrathecal mesenchymal stem cells in patients with multiple sclerosis: a pilot study with booster injection. Immunol Invest.

[61] Guliyeva G, Guzman RAT, Verduzco FRV, Akinduro OO, Guerrero-Cazares H, Meade PS, et al. Use of mesenchymal stem cells in pre-clinical models of spinal cord injury. Paraplegia. 2021;1–16.

[62] Yamout B, Hourani R, Salti H, Barada W, El-Hajj T, Al-Kutoubi A (2010). Bone marrow mesenchymal stem cell transplantation in patients with multiple sclerosis: a pilot study. J Neuroimmunol.

[63] Detiger SE, Helder MN, Smit TH, Hoogendoorn RJ (2015). Adverse effects of stromal vascular fraction during regenerative treatment of the intervertebral disc: observations in a goat model. Eur Spine J.

[64] Chen NF, Sung CS, Wen ZH, Chen CH, Feng CW, Hung HC (2018). Therapeutic effect of platelet-rich plasma in rat spinal cord injuries. Front Neurosci.

[65] El-Seddawy FD, Samy MTM, Mekkawy NHM, Behery AES, Youssef WOM (2020). Experimental trials of spinal cord injury treatment in rats. J Anim Heal Prod.

[66] Vikartovska Z, Kuricova M, Farbakova J, Liptak T, Mudronova D, Humenik F (2020). Stem cell conditioned medium treatment for canine spinal cord injury: pilot feasibility study. Int J Mol Sci.

[67] Song YY, Peng CG, Ye XB (2015). Combination of edaravone and neural stem cell transplantation repairs injured spinal cord in rats. Genet Mol Res.

[68] Haist V, Spitzbarth I, Bock P, Beineke A, Wewetzer K, Baumgärtner W (2010). Morphological characterization of traumatic spinal cord injury caused by intervertebral disc disease in dogs. J Comp Pathol.

[69] Gomes-leal W, Corkill DJ, Picanc CW (2005). Systematic analysis of axonal damage and inflammatory response in different white matter tracts of acutely injured rat spinal cord. Brain Res.

[70] Abdallah AN, Shamaa AA, El-Tookhy OS (2020). Ethidium bromide induced demyelination of the central nervous system in a dog model of secondary progressive multiple sclerosis. J Curr Vet Res.

[71] Al-Karim S, Ramadan WS, Abdel-Hamid GA, Al QF (2019). Does neuroectodermal stem cells transplantation restore neural regeneration and locomotor functions in compressed spinal cord injury rat model?. Int J Morphol.

[72] Hedayatpour A, Ragerdi I, Pasbakhsh P, Kafami L, Atlasi N, Mahabadi VP (2013). Promotion of remyelination by adipose mesenchymal stem cell transplantation in a cuprizone model of multiple sclerosis. Cell J.

[73] Ganji R, Razavi S, Ghasemi N, Mardani M (2020). Improvement of remyelination in demyelinated corpus callosum using human adipose-derived stem cells (hADSCs) and pregnenolone in the cuprizone rat model of multiple sclerosis. J Mol Neurosci.

[74] Wegener A, Deboux C, Bachelin C, Frah M, Kerninon C, Seilhean D (2015). Gain of Olig2 function in oligodendrocyte progenitors promotes remyelination. Brain.

[75] Yeung MS, Djelloul M, Steiner E, Bernard S, Salehpour M, Possnert G (2019). Oligodendrocyte generation dynamics in multiple sclerosis Maggie. Nature.

[76] Zuk P, Zhu M, Ashjian P, Ugarte D, Huang J, Mizuno H (2002). Human adipose tissue is a source of multipotent stem cells. Mol Biol Cell.

[77] Ghasemi N (2018). Transdifferentiation of human adipose-derived mesenchymal stem cells into oligodendrocyte progenitor cells. Iran J Neurol.

[78] Fushimi S, Shirabe T (2002). The reaction of glial progenitor cells in remyelination following ethidium bromide-induced demyelination in the mouse spinal cord. Neuropathol.

[79] El-Habta R, Sloniecka M, Kingham PJ, Backman LJ (2018). The adipose tissue stromal vascular fraction secretome enhances the proliferation but inhibits the differentiation of myoblasts. Stem Cell Res Ther.

[80] El-Habta R, Andersson G, Kingham PJ, Backman LJ (2021). Anti-apoptotic effect of adipose tissue-derived stromal vascular fraction in denervated rat muscle. Stem Cell Res Ther.

[81] Wang Z-H, Xiang J, Liu X, Yu SP, Manfredsson FP, Sandoval IM (2019). Deficiency in BDNF/TrkB neurotrophic activity stimulates δ-secretase by upregulating C/EBPβ in Alzheimer’s disease. Cell Rep.

[82] Johnson S, Liston C (2021). MeCP2 for sustained antidepressant effects. Nat Neurosci.

[83] Gao L, Zhang Y, Sterling K, Song W (2022). Brain-derived neurotrophic factor in Alzheimer's disease and its pharmaceutical potential. Transl neurodegener.

[84] Kim JW, Autry AE, Na ES, Adachi M, Bjorkholm C, Kavalali ET (2021). Sustained effects of rapidly acting antidepressants require BDNF-dependent MeCP2 phosphorylation. Nat Neurosci.

[85] Li H, Shang J, Zhang C, Lu R, Chen J, Zhou X (2020). Repetitive transcranial magnetic stimulation alleviates neurological deficits after cerebral ischemia through interaction between RACK1 and BDNF exon IV by the phosphorylation-dependent factor MeCP2. Neurother.

[86] Bowles AC, Wise RM, Gerstein BY, Thomas RC, Ogelman R, Manayan RC (2018). Adipose stromal vascular fraction attenuates TH1 cell-mediated pathology in a model of multiple sclerosis. J Neuroinflamm.

[87] Duma C, Kopyov O, Kopyov A, Berman M, Lander E, Elam M (2019). Human intracerebroventricular (ICV) injection of autologous, non-engineered, adipose-derived stromal vascular fraction (ADSVF) for neurodegenerative disorders: results of a 3-year phase 1 study of 113 injections in 31 patients. Mol Biol Rep.

[88] Tang YL, Zhao Q, Qin X, Shen L, Cheng L, Ge J (2005). Paracrine action enhances the effects of autologous mesenchymal stem cell transplantation on vascular regeneration in rat model of myocardial infarction. Ann Thorac Surg.

[89] Shabbir A, Cox A, Rodriguez-Menocal L, Salgado M, Van Badiavas E (2015). Mesenchymal stem cell exosomes induce proliferation and migration of normal and chronic wound fibroblasts and enhance angiogenesis in vitro. Stem Cells Dev.

[90] Minnone G, De Benedetti F, Bracci-Laudiero L (2017). NGF and Its receptors in the regulation of inflammatory response. Int j mol sci.

[91] Zhuang WZ, Lin YH, Su LJ, Wu MS, Jeng HY, Chang HC (2021). Mesenchymal stem/stromal cell-based therapy: mechanism, systemic safety and biodistribution for precision clinical applications. J Biomed Sci.

[92] Jaramillo-Merchan J, Jones J, Ivorra JL, Pastor D, Viso-León MC, Armengól JA (2013). Mesenchymal stromal-cell transplants induce oligodendrocyte progenitor migration and remyelination in a chronic demyelination model. Cell Death Dis.

[93] Liu GY, Wu Y, Kong FY, Ma S, Fu LY, Geng J (2021). BMSCs differentiated into neurons, astrocytes and oligodendrocytes alleviated the inflammation and demyelination of EAE mice models. PLoS ONE.

[94] Li S, Guan H, Zhang Y, Li S, Li K, Hu S (2021). Bone marrow mesenchymal stem cells promote remyelination in spinal cord by driving oligodendrocyte progenitor cell differentiation via TNFα/RelB-Hes1 pathway: a rat model study of 2, 5-hexanedione-induced neurotoxicity. Stem Cell Res Ther.

