# Supplementary material for: Laser-activated autologous adipose tissue-derived stromal vascular fraction restores spinal cord architecture and function in multiple sclerosis cat model
Source: Stem Cell Res Ther. 2023 Jan 11;14:6. doi: 10.1186/s13287-022-03222-2 (PMC9832640; doi:10.1186/s13287-022-03222-2)
Supplement: Supplementary file 1 — Additional file 1. Supplementary figures. [file 13287_2022_3222_MOESM1_ESM.docx]

**Supplementary information**

*Ms. Ref. No.:* SCRT-D-22-00881

*Title:* Laser activated autologous adipose tissue-derived stromal vascular fraction restore spinal cord architecture and function in multiple sclerosis Cat model

*Journal: Stem Cell Research & Therapy*

**Farid et. al.,**


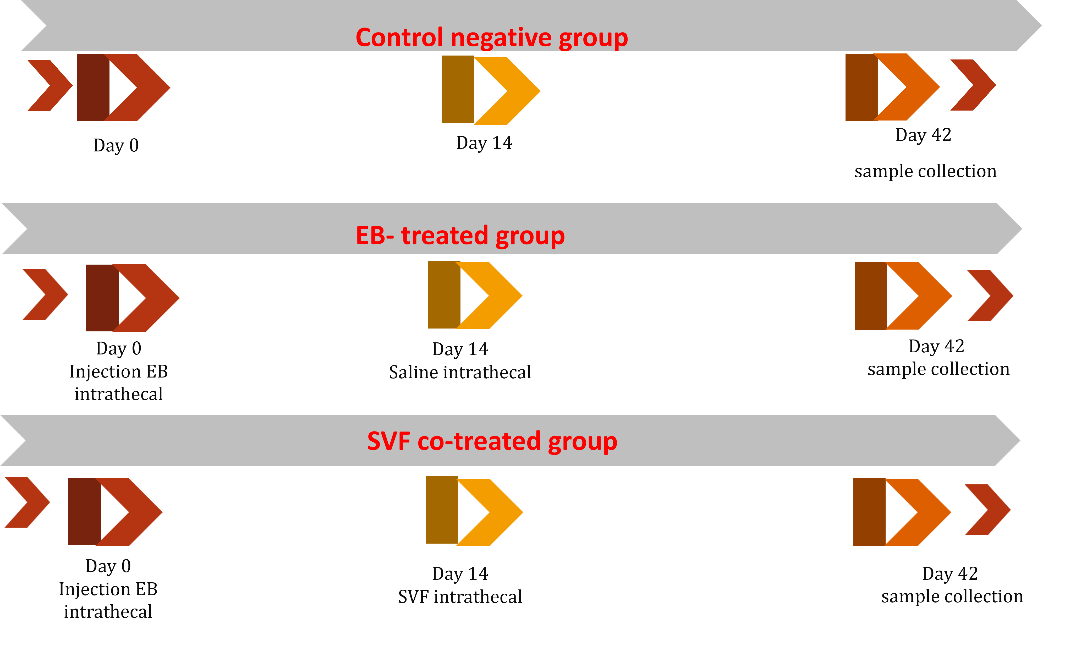


Fig. (1) Time line of the study


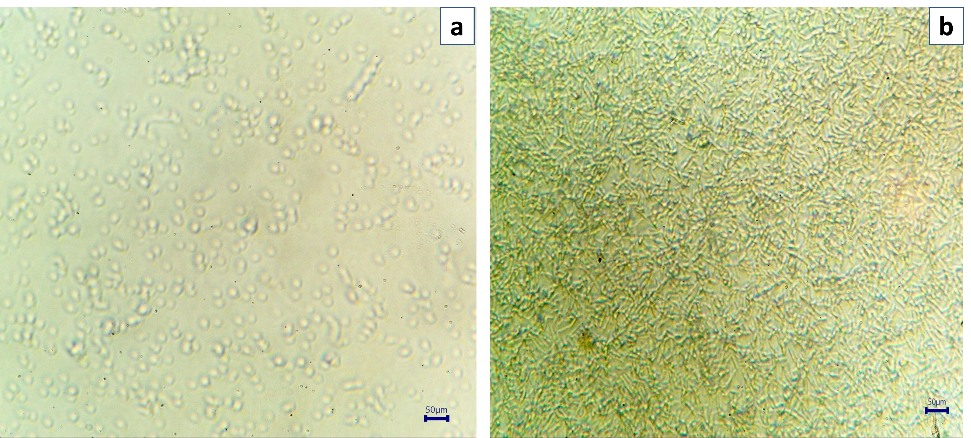


Fig. (2) ADMSCs morphology A) 3 days’ culture 20x, B) 10 days’ culture 4x (scale bars, 50µm)


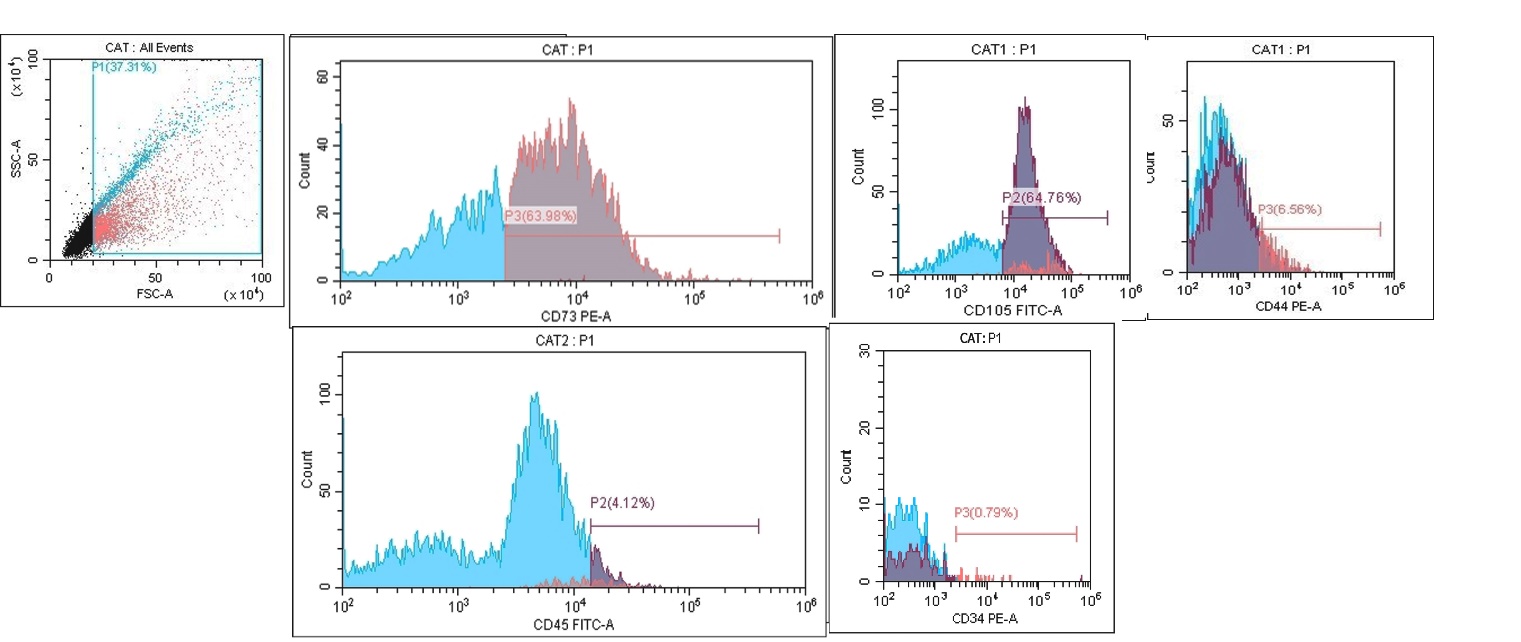


Fig. (3) Flow cytometry analysis of ADMSCs at passage 3 against surface markers CD34,CD44, CD73, CD105 and CD45.


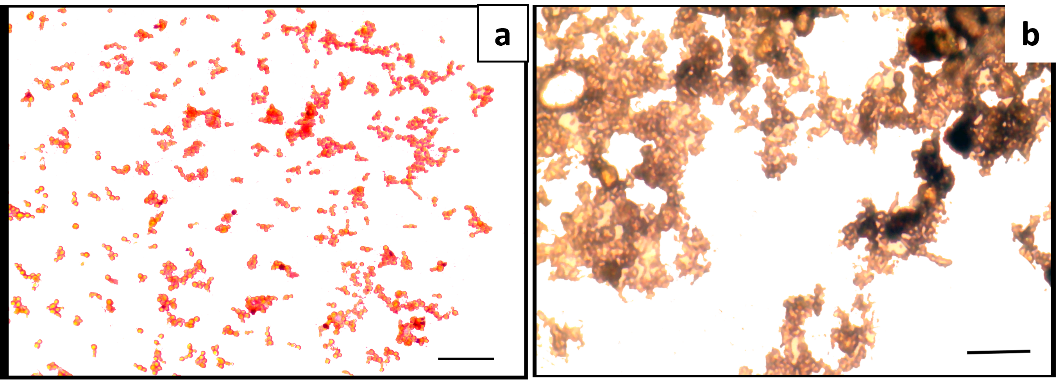


Fig. (4) ADMSCs differentiation a) Chondrogenic differentiation, Safranin-O staining 4x. b) Adipogenic differentiation, oil red O staining 4x (Scale bars 100µm).
